# Lipid Metabolism Reprogramming in Diffuse Large B-Cell Lymphoma (DLBCL): Mechanisms and Treatment Strategies

**DOI:** 10.3390/cancers18040701

**Published:** 2026-02-20

**Authors:** Yue-E Ding, Yi-Ran Zhong, Lai-Shun Zhang, Lei Xu, Jia Li, Yi Wen

**Affiliations:** 1 Department of Pharmacy, Zhongshan City People’s Hospital, Zhongshan 528403, China; dingyuee1876@zidd.ac.cn (Y.-E.D.); z13461166364@outlook.com (L.-S.Z.); 2 Zhongshan Institute for Drug Discovery, Shanghai Institute of Materia Medica, Chinese Academy of Sciences, Zhongshan 528400, China; zhongyiran23@mails.ucas.ac.cn (Y.-R.Z.); 0024xl@zidd.ac.cn (L.X.); 3 School of Pharmaceutical Science and Technology, Hangzhou Institute for Advanced Study, University of Chinese Academy of Sciences, Hangzhou 310024, China; 4 University of Chinese Academy of Sciences, Beijing 100049, China

**Keywords:** diffuse large B-cell lymphoma, lipid metabolism reprogramming, tumor immune microenvironment, ferroptosis, prognostic biomarkers, targeted drugs, treatment strategies

## Abstract

Diffuse large B-cell lymphoma (DLBCL) is a common, aggressive non-Hodgkin lymphoma with significant molecular heterogeneity. This variability arises in part from its distinct molecular subtypes, including germinal center B-cell-like (GCB) DLBCL, activated B-cell-like (ABC) DLBCL, and primary mediastinal B-cell lymphoma (PMBL), which differ markedly in their genetic profiles, signaling pathway activities, and clinical outcomes. Although rituximab-based R-CHOP regimens have significantly improved patient outcomes, around 40% of patients still experience relapsed or refractory disease. DLBCL cells sustain their rapid proliferation through the establishment of an intricate lipid metabolism regulatory network. The interplay between this network, cell death mechanisms (e.g., ferroptosis), and the tumor immune microenvironment (TIME) significantly impacts the malignant progression of the disease and its resistance to treatment. This review summarizes recent advances in understanding the molecular mechanisms and interplay among these processes in DLBCL and discusses the clinical relevance of associated prognostic biomarkers, thus providing new insights into the development of precision therapies.

## Simple Summary

Diffuse large B-cell lymphoma (DLBCL) is a highly heterogeneous and aggressive malignancy, in which relapse and therapeutic resistance remain major clinical challenges. Growing evidence indicates that lipid metabolism reprogramming serves as a central driver of DLBCL progression, treatment resistance, and immune evasion. Aberrant fatty acid, cholesterol, and phospholipid metabolism promotes tumor growth, suppresses ferroptosis, and remodels the tumor immune microenvironment (TIME). Key metabolic enzymes and pathways, including FASN, FAO-related enzymes, the PI3K–AKT–mTOR signaling axis, and ceramide metabolism, are closely associated with ferroptosis susceptibility and immune cell function. Furthermore, metabolic crosstalk between lymphoma cells and immune components, such as macrophages and T cells, contributes to immunosuppression and resistance to immunotherapy. This review summarizes recent advances in lipid metabolism reprogramming, ferroptosis regulation, and TIME remodeling in DLBCL, and outlines prognostic biomarkers and therapeutic strategies targeting lipid metabolism, ferroptosis, and the tumor immune microenvironment, thereby providing new insights for improving treatment outcomes.

## 1. Introduction

Diffuse large B-cell lymphoma (DLBCL) is the most common lymphoid malignancy and a major subtype of non-Hodgkin lymphoma (NHL). Over 50% of patients with DLBCL achieve remission with the first-line rituximab, cyclophosphamide, doxorubicin, vincristine, and prednisone (R-CHOP) regimen; however, around 40% of patients eventually relapse and have a poor prognosis [1,2]. Recent studies have demonstrated that the initiation and progression of diffuse large B-cell lymphoma (DLBCL) are closely associated with lipid metabolic reprogramming. As a novel form of regulated cell death dependent on lipid peroxidation, ferroptosis and its crosstalk with lipid metabolism disorders in DLBCL have emerged as a research hotspot in tumor targeted therapy.

Based on cell-of-origin (COO) analysis and gene expression profiling (GEP), DLBCL was initially classified into two subtypes, namely germinal center B-cell-like (GCB) and activated B-cell-like (ABC) DLBCLs [3]. A third subtype, primary mediastinal B-cell lymphoma (PMBL), was later identified through GEP [4]. PMBL typically occurs at a median age of 33 and is more common in women, with 35% of cases occurring in women under 35 years of age. It has approximately a 64% 5-year survival rate, which is significantly higher than those of ABC-DLBCL (30%) and GCB-DLBCL (59%). PMBL commonly involves intrathoracic structures (lungs/breasts), while other DLBCL subtypes more frequently affect extrathoracic sites [5,6]. Recent transcriptomic studies have further classified diffuse large B-cell lymphoma (DLBCL) into four subtypes—germinal center-like (GC-like), mesenchymal (MS), inflammatory (IN), and depleted (DP)—based on the molecular characteristics of the tumor microenvironment (TME). The prognostic differences among these subtypes are closely associated with immune cell infiltration, stromal components, and molecular expression profiles within the TME [7]. Regardless of the classification system used, stratifying DLBCL by molecular subtype is essential for guiding targeted therapeutic strategies and improving clinical outcomes. 

The NF-κB and PI3K-Akt-mTOR signaling pathways are commonly dysregulated in DLBCL and contribute to its pathogenesis, often involving genetic alterations in key regulators, such as MYC, EZH2, and MYD88 [8]. Notably, the pathogenic signaling pathways and genetic alterations vary substantially across the DLBCL subtypes. For example, ABC-DLBCL is typically more aggressive and associated with a poorer prognosis compared to GCB-DLBCL. ABC-DLBCL also shows reduced response to the standard R-CHOP regimen and has a high relapse rate [3]. Furthermore, in ABC-DLBCL, the NF-κB pathway and its downstream kinase BTK are constitutively activated by the B-cell receptor (BCR), promoting tumor cell survival and proliferation [8]. In contrast, the GCB-DLBCL is characterized by EZH2 mutations, constitutive activation of the PI3K-AKT-mTOR pathway, and overexpression of BCL2 and BCL6. R-CHOP is the first-line regimen for GCB-DLBCL. Despite a generally good response in most patients, some patients experience relapse [9]. PMBL also exhibits characteristic NF-κB activation; however, it is associated with more favorable clinical outcomes compared to ABC-DLBCL [6]. Therefore, targeted interventions directed at subtype-specific molecular pathways are critical to improving patient outcomes. Moreover, developing novel therapeutic strategies to enhance remission rates, particularly in refractory and relapsed cases, represents a critical unmet need in DLBCL.

The etiology of DLBCL is closely linked to genetic, environmental, and lifestyle factors, with obesity representing a major risk factor [10]. The widespread adoption of high-sugar and high-fat diets has caused a steady increase in obesity, with more than two billion individuals worldwide classified as overweight, accounting for approximately 30% of the global population. Obesity is further associated with increased mortality risk across multiple malignancies, including DLBCL [11,12,13]. A Japanese case–control study demonstrated that early adulthood obesity is significantly associated with an increased risk of diffuse large B-cell lymphoma (DLBCL) [14]. In contrast, another study involving U.S. veterans found that the risk of mortality was significantly lower in overweight (BMI 25–30) or obese (BMI ≥ 30) patients than in those with normal weight (hazard ratios [HRs] of 0.73 and 0.68, respectively) [15]. These findings suggest that a higher BMI may exert a certain protective effect on the survival of DLBCL patients. This phenomenon may be related to obesity-mediated lipid metabolism disorders and altered sensitivity to ferroptosis. Obesity can regulate lipid metabolic reprogramming by upregulating insulin resistance and inflammatory factors, and DLBCL cells may gain a survival advantage by adapting to the lipid metabolic environment and regulating the expression of key ferroptosis-related molecules, whose underlying mechanisms require further investigation. In addition, metabolic disorders themselves can promote lymphoma development by affecting the tumor immune microenvironment (TIME) [16]; as a core component of metabolic reprogramming, the crosstalk between lipid metabolism and ferroptosis may further exacerbate the heterogeneity of DLBCL. 

In view of this, this review systematically summarizes the relationship between lipid metabolic reprogramming and the development and progression of diffuse large B-cell lymphoma (DLBCL). Its core innovation lies in, for the first time, using ferroptosis regulation as a central link to systematically integrate the interactive network among lipid metabolic reprogramming, ferroptosis, and the tumor immune microenvironment (TIME), thereby overcoming the limitations of previous single-dimensional studies. By focusing on the subtype-specific metabolic vulnerabilities of DLBCL and using ferroptosis sensitivity as a basis for therapeutic stratification, it aims to provide a novel integrative perspective and potential targets for optimizing treatment strategies and improving the prognosis of patients with relapsed/refractory DLBCL.

## 2. Lipid Metabolism Reprogramming in DLBCL

Metabolic reprogramming is a hallmark of cancer cells [17]. In DLBCL, the reprogramming of lipid metabolism plays a critical role in enabling cells to sustain rapid proliferation and adapt to microenvironmental stress [18]. This process is characterized by aberrant activation of fatty acid (FA) synthesis (FAS), FA oxidation (FAO), and cholesterol metabolism, and is mediated through the coordinated regulation of key enzymes and signaling pathways. The dysregulation of lipid metabolism is a central mechanism driving tumor development and progression and represents a major focus of current cancer research [19].

### 2.1. FA Metabolism in DLBCL

**Figure 1:**
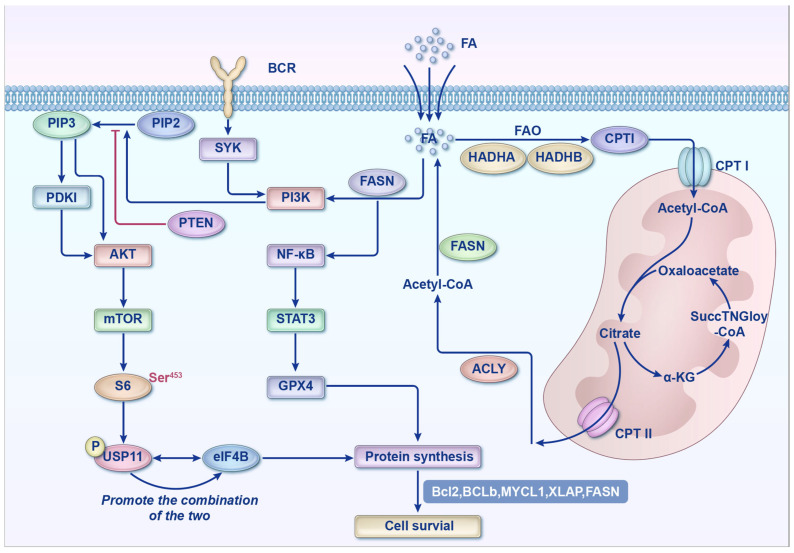
Molecular Mechanisms of Lipid Metabolic Reprogramming and Associated Cell Survival Signaling Pathways in DLBCL. This schematic illustrates the key pathways linking BCR signaling, lipid metabolic reprogramming, ferroptosis regulation, and anti-apoptotic mechanisms in DLBCL. B-cell receptor (BCR) activation triggers SYK signaling, leading to PI3K–AKT–mTOR pathway activation. PI3K phosphorylates PIP2 to generate PIP3, which activates AKT via PDK1. AKT then activates mTOR, promoting protein synthesis and cell survival through phosphorylation of S6 and translation initiation factors such as eIF4E.Concurrently, key enzymes including FASN (fatty acid synthase) and ACLY (ATP-citrate lyase) promote acetyl-CoA production. Acetyl-CoA serves as a central node in lipid metabolism, energy metabolism (e.g., TCA cycle), and protein acetylation.STAT3 (signal transducer and activator of transcription 3) transcriptionally regulates GPX4 (glutathione peroxidase 4), a key inhibitor of ferroptosis that prevents lipid peroxide accumulation. Dysregulation of this pathway may lead to ferroptosis, impacting DLBCL cell survival. Ultimately, anti-apoptotic proteins such as BCL2, BCL6, and XIAP synergistically promote cell survival. Hydroxyacyl-CoA dehydrogenase trifunctional multienzyme complex subunit alpha (HADHA), a key enzyme in FAO, is highly expressed in 68% of DLBCL cases and is strongly associated with poor prognosis. HADHB forms a heterodimer with HADHA, functioning as the FAO enzyme complex. Studies have shown that *HADHB* knockout suppresses the proliferation of DLBCL cells, while ranolazine, an FAO inhibitor, induces cell death. This effect can be reversed by ferrostatin-1, indicating that FAO promotes DLBCL progression by inhibiting ferroptosis [29]. Furthermore, Montagne et al. [30] conducted metabolic phenotyping of DLBCL cell lines and demonstrated that targeting FA metabolism induced cell death regardless of the DLBCL metabolic subtype (oxidative phosphorylation [OXPHOS] or B-cell receptor [BCR]/glycolytic); this cytotoxic effect was significantly enhanced when combined with mTOR inhibitors. Importantly, FAs contribute to resistance against metformin and L-asparaginase in DLBCL cells by mitigating mitochondrial stress, suggesting that FA metabolism may be a key target to overcome resistance to metabolic agents. Therefore, targeting FA metabolism-related signaling pathways represents a promising therapeutic strategy for DLBCL.

DLBCL cells sustain energy production and membrane biosynthesis through enhanced FAS and FAO. FA synthase (FASN), the rate-limiting enzyme in de novo FAS, regulates biological functions via the p-ERK/BCL-2 signaling pathway. FASN is highly expressed in DLBCL, particularly in MYC/BCL2 double-positive lymphomas. The FASN expression is strongly associated with a more aggressive phenotype. Therefore, FASN is regarded as a potential therapeutic target in DLBCL [20]. FASN also activates the PI3K-S6K signaling pathway by regulating ubiquitin-specific protease 11 (USP11), which promotes its interaction with eukaryotic initiation factor 4B (eIF4B) and sustains aberrant protein translation in tumor cells [21]. The PI3K activation leads to the conversion of phosphatidylinositol 4,5-bisphosphate (PIP2) into phosphatidylinositol 3,4,5-trisphosphate (PIP3). PIP3 then recruits proteins containing pleckstrin homology domains (PHDs), resulting in the activation of 3-phosphoinositide-dependent protein kinase 1 (PDK1). PDK1 subsequently phosphorylates AKT at threonine 308 (T308), enabling AKT to phosphorylate the downstream substrates that promote cell survival and metabolism [22,23,24,25,26]. The tumor suppressor gene *PTEN* contributes to the pathogenesis of DLBCL by dephosphorylating PIP3 to PIP2 through its lipid phosphatase activity, thereby negatively regulating the PI3K/AKT pathway [27]. In addition, FASN inhibits ferroptosis via the NF-κB/STAT3/GPX4 axis, resulting in resistance to adriamycin (ADM) [28] (Figure 1). These findings suggest that FASN may represent a key mediator linking lipid synthesis with resistance to cell death. 

### 2.2. Cholesterol Metabolism in DLBCL

Abnormal cholesterol metabolism plays a critical role in DLBCL proliferation and signaling. Activation of spleen tyrosine kinase (SYK) via BCR signaling stimulates downstream pathways, including PI3K/AKT and NF-κB, maintains cholesterol biosynthesis, and preserves BCR integrity within lipid rafts. SYK inhibition disrupts this process and induces apoptosis in BCR-dependent DLBCL [31]. In addition, toosendanin (TSN), a triterpene compound, reduces the mRNA and protein of polo-like kinase 1 (PLK1) by downregulating the PI3K/AKT signaling pathway, thereby inducing DLBCL cell apoptosis, cell cycle arrest, and cell death. Transcriptomic analysis indicated that TSN could upregulate cholesterol biosynthesis in these cells, and combination treatment with statins further enhanced the anti-tumor activity of TSN while reducing toxicity [32]. 

Cholesterol efflux is a key mechanism of immunosuppression in the TME. In primary refractory DLBCL, M2 macrophages inhibit CD8^+^ T cell cytotoxicity through cholesterol efflux, thereby contributing to resistance to CAR-T cell therapy [33]. This process is accompanied by the activation of immune checkpoint molecules (such as LGALS9-HAVCR2 and CTLA4) and targeting cholesterol metabolism may help overcome resistance to immunotherapy. Therefore, cholesterol metabolism represents a potential regulatory target for the treatment of DLBCL.

### 2.3. Phospholipid Metabolism in DLBCL

Abnormal phospholipid metabolism plays a critical role in DLBCL proliferation, signaling, and therapy resistance. Phospholipids serve as core components of cellular membranes, and their metabolic reprogramming is closely associated with the malignant phenotype of DLBCL. The analysis of DLBCL lymph node samples using gas cluster ion beam secondary ion mass spectrometry (GCIB-SIMS) demonstrated that samples with a high proliferation index exhibited distinct phospholipid signatures [34]. Mass spectrometry imaging of R-CHOP-resistant DLBCL revealed specific lipid profile abnormalities, including significant increases in phosphatidylinositol and sphingomyelin fragments [35]. This suggested that dysregulation of phospholipid metabolism may be a key mechanism underlying resistance to standard chemotherapy in DLBCL. In addition, a recent plasma lipidomics study demonstrated that female DLBCL patients had significantly altered levels of lysophosphatidylinositol, sphingomyelin, and phosphatidylinositol. Accumulation of arachidonic acid (AA) lipoxygenase pathway products was closely associated with tumor development, suggesting that phospholipid remodeling and inflammation-associated lipid signaling play a critical role in DLBCL [36].

The intermediates of phospholipid metabolism are involved in regulating signaling pathways associated with DLBCL. Lysine deacetylase inhibitors (KDACIs) induce a novel dependence of DLBCL cells on the choline pathway, a key pathway for phospholipid synthesis, while simultaneously activating PI3K signaling. Notably, the concomitant use of choline pathway inhibitors significantly enhances the antitumor effect of KDACIs, indicating that phospholipid metabolism influences DLBCL progression by cross-regulating PI3K and other signaling pathways [37]. In addition, diacylglycerol (DAG), a key intermediate of phospholipid metabolism, functions as a lipid second messenger and regulates cell proliferation and signaling through effectors, such as protein kinase C (PKC) and protein kinase D (PKD) [38], thereby driving DLBCL development. 

Aberrant phospholipid metabolism may also influence the interaction of DLBCL cells with the TME. Liposomes are used for targeted drug delivery and contain lecithin, which, upon fusion with macrophage membranes, markedly enhances drug internalization and accumulation in DLBCL cells [39]. These findings suggest that the recognition of specific phospholipid structures by DLBCL cells may affect their interactions with microenvironmental components, modulating drug sensitivity. Thus, targeting phospholipid metabolism may represent a potential strategy to improve treatment efficacy in DLBCL.

### 2.4. Regulatory Network for Lipid Metabolic Reprogramming

Lipid metabolic reprogramming is regulated by multiple signaling pathways and epigenetic mechanisms. The PI3K/AKT/mTOR pathway plays a central role by promoting FASN-mediated FAS by activating S6K and enhancing protein translation via the USP11-eIF4B axis [21]. Additionally, histone deacetylase inhibitors (HDACIs), such as Panobinostat, can remodel lipid metabolism through the choline pathway, thereby activating PI3K signaling and diminishing their antitumor effect; this effect can be reversed by combining HDACIs with choline pathway inhibitors [37]. These findings suggest that cancer cells may acquire resistance to epigenetic therapies through metabolic adaptation. 

Furthermore, metabolites generated by metabolic processes also participate in regulatory mechanisms, which has become a research hotspot in recent years. One study demonstrated that YTHDF2, an m6A-binding protein, is highly expressed in DLBCL and recognizes the m6A-modified site on alkaline ceramidase 2 (*ACER2*) mRNA. This interaction enhances ACER2 mRNA stability and protein expression, thereby promoting the hydrolysis of ceramide into sphingosine and activating the PI3K/AKT pathway, ultimately driving tumor proliferation. The discovery of the YTHDF2-ACER2-ceramide axis provides a potential new avenue for targeted therapy in DLBCL [40]. α-Ketoglutarate (α-KG), an intermediate of glutamine metabolism, inhibits DLBCL growth by inducing reactive oxygen species (ROS) production and TP53-mediated ferroptosis [41]. FAs contribute to resistance against metformin and L-asparaginase by alleviating mitochondrial stress [30]. Therefore, a cross-regulation between lipid metabolism and other metabolic pathways is a key mechanism in DLBCL pathogenesis.

## 3. Multi-Level Regulatory Network of Ferroptosis in DLBCL

**Figure 2:**
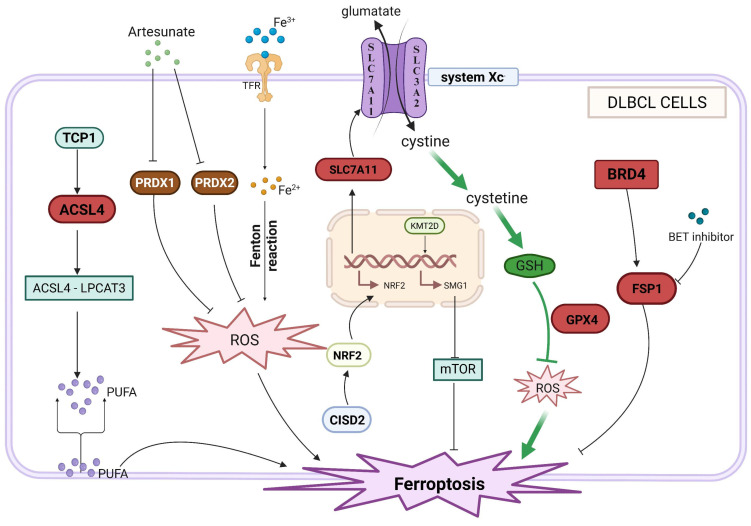
Multilevel Regulatory Network of Ferroptosis in Diffuse Large B-Cell Lymphoma. Ferroptosis in DLBCL is regulated through antioxidant defense, iron metabolism, epigenetic signaling, and lipid peroxidation. The transsulfuration pathway generates reduced glutathione (GSH), which activates GPX4 to reduce lipid peroxides and inhibit ferroptosis. CISD2 reinforces this defense via the NRF2/SLC7A11/GPX4 axis, while its loss promotes ferroptosis. Intracellular Fe^3+^ is reduced to Fe^2+^, notably by GSH, driving the Fenton reaction and lipid peroxidation. Artesunate (ART) exacerbates ROS by inhibiting PRDX1/2. Epigenetically, KMT2D enhances ferroptosis sensitivity via H3K4me1-dependent SMG1 activation and mTOR suppression, whereas BRD4 upregulates FSP1; BET inhibitors counteract this, sensitizing GCB-subtype cells. In lipid metabolism, TCP1 stabilizes ACSL4, activating ACSL4/LPCAT3 signaling to incorporate PUFAs into phospholipids and amplify peroxidation, facilitating ferroptosis.

In 2012, Dixon first proposed the concept of ferroptosis, a novel form of cell death distinct from apoptosis, necrosis, and autophagy in terms of morphology and function. This process is characterized by the iron-dependent accumulation of lipid peroxides, with phospholipid peroxidation serving as a key step, meticulously regulated by multiple signaling pathways that collectively determine the progression of ferroptosis [42,43]. In cancer research, ferroptosis has been implicated in the development and progression of various tumors [44,45,46]. Notably, early studies found that many lymphoma cells are susceptible to ferroptosis, particularly in Diffuse Large B-Cell Lymphoma (DLBCL), where ferroptosis plays a critical role, and DLBCL cells exhibit significantly higher sensitivity compared to other tumors. This susceptibility is under complex regulation by pathways involving lipid metabolism, epigenetics, and others (Figure 2). Therefore, using ferroptosis inducers to treat DLBCL is undoubtedly a promising new direction. Based on this finding, we summarize the specific regulatory mechanisms underlying ferroptosis susceptibility in DLBCL, aiming to provide new insights for DLBCL treatment.

### 3.1. Regulation of Cystine Uptake and the GSH/GPX4 Antioxidant Defense Axis

The antioxidant defense system represents a core regulatory layer in ferroptosis, among which glutathione peroxidase 4 (GPX4) reduces lipid peroxides by consuming glutathione (GSH), forming a critical barrier against ferroptosis [47]. The antioxidant defense of diffuse large B-cell lymphoma (DLBCL) cells relies on specific cystine acquisition pathways. Some DLBCL cell lines are unable to synthesize cysteine from methionine via the transsulfuration pathway [48], and must depend on System Xc^−^ (with core subunits SLC7A11/xCT) to uptake extracellular cystine. This cystine is subsequently used for GSH synthesis to maintain GPX4 activity, rendering such cells highly sensitive to System Xc^−^ inhibitors [48,49,50,51,52].

As a key upstream molecule in this regulatory axis, CISD2 enhances cellular antioxidant capacity by activating the NRF2/SLC7A11/GPX4 signaling pathway. Specifically, CISD2 promotes the nuclear translocation of NRF2, upregulates SLC7A11 expression to increase cystine uptake and glutathione (GSH) synthesis, and ultimately maintains GPX4 activity to inhibit ferroptosis [52,53]. High CISD2 expression is closely associated with tumor progression in diffuse large B-cell lymphoma (DLBCL), whereas CISD2 silencing significantly reduces GPX4 levels, disrupts antioxidant homeostasis, and induces ferroptosis [52]. In addition, the antioxidant peroxidase peroxiredoxin (PRDX) family is also involved in this regulatory hierarchy. Artesunate (ART) can directly bind to the Gly4 residue of PRDX1 and the Arg7/Thr120 residues of PRDX2, inhibiting their reactive oxygen species (ROS)-scavenging function. This leads to the accumulation of lipid peroxides and synergistically promotes ferroptosis, suggesting that the PRDX family may serve as novel therapeutic targets for ferroptosis regulation in DLBCL [54].

### 3.2. Regulation of Iron Homeostasis

Ferroptosis relies on the accumulation of the intracellular labile iron pool (LIP), making iron homeostasis a critical regulatory layer in this process. Iron oxide nanoparticles (IONs) can promote ferroptosis by coordinately upregulating transferrin receptor (TFR) and downregulating ferroportin (FPN), thereby elevating LIP levels. This leads to the generation of abundant reactive oxygen species (ROS) via the Fenton reaction, ultimately triggering ferroptosis [54].

Furthermore, ferritin heavy chain 1 (FTH1), the primary intracellular iron storage protein, mediates ferroptosis through the process of ferritinophagy. Loss of SH3GL1 relieves its inhibition on ferritinophagy, promotes FTH1 degradation and subsequent release of labile iron, thereby increasing cellular susceptibility to ferroptosis [55]. Conversely, high SH3GL1 expression inhibits doxorubicin-induced ferroptosis, contributing to chemotherapy resistance in DLBCL. Clinical data confirm that high SH3GL1 expression is significantly associated with poor patient prognosis, highlighting its potential as a biomarker for both ferroptosis tolerance and chemotherapy resistance [55]. In addition, prostaglandin D2 synthase (PTGDS) can bind to heme oxygenase 1 (HMOX1), inhibiting HMOX1-mediated heme degradation and subsequent ferritinophagy, which reduces labile iron accumulation. This interplay offers a novel perspective on the cross-regulation between iron metabolism and heme metabolism in DLBCL [56].

### 3.3. Regulation of Key Signaling Pathways

Multiple cellular signaling pathways form an additional regulatory layer by integrating metabolic, antioxidant, and lipid synthesis signals. The NF-κB/STAT3 pathway suppresses ferroptosis through the crosstalk between lipid metabolism and antioxidant molecules. As a downstream target of this pathway, fatty acid synthase (FASN) promotes the transcriptional activation of GPX4 via the NF-κB/STAT3 signaling axis. This not only enhances cellular antioxidant capacity but also mediates doxorubicin resistance, ultimately inhibiting ferroptosis [28]. Within lipid metabolism-related pathways, acyl-CoA synthetase long-chain family member 4 (ACSL4) facilitates the activation of polyunsaturated fatty acids (PUFAs). These activated PUFAs are subsequently incorporated into membrane phospholipids via the LPCAT3 pathway, providing ample substrate for lipid peroxidation [57]. TCP1, a regulatory factor of this pathway, interacts with ACSL4 and reduces its ubiquitin-mediated degradation, thereby activating the ACSL4/LPCAT3 axis and enhancing the cytotoxic effect of the ferroptosis inducer RSL3 [57].

### 3.4. Epigenetic Regulation

Epigenetic mechanisms modulate the transcriptional expression of ferroptosis-associated genes, demonstrating subtype specificity in DLBCL. Histone-modifying enzymes serve as core regulators within this layer. The histone methyltransferase KMT2D enhances the susceptibility of DLBCL cells to ferroptosis by activating SMG1 gene transcription through H3K4me1 modification, which in turn antagonizes the mTOR signaling pathway [58]. Conversely, the bromodomain protein BRD4 protects germinal center B-cell (GCB) subtype DLBCL cells from ferroptosis by sustaining the transcriptional expression of the ferroptosis suppressor protein FSP1 [59]. BET inhibitors can specifically downregulate BRD4-mediated FSP1 expression, thereby sensitizing the GCB subtype to ferroptosis inducers and offering a potential target for subtype-specific therapy [59].

## 4. Remodeling of Tumor Immune Microenvironment

The tumor immune microenvironment (TIME) of diffuse large B-cell lymphoma (DLBCL) is highly heterogeneous, with the functional states and spatial distribution of its cellular components dynamically varying across tumor subtypes and stages of disease progression. Metabolic reprogramming of both tumor cells and immune cells represents a central link in their reciprocal interactions. Metabolites not only regulate immune cell polarization and effector functions but also influence tumor cell survival and susceptibility to ferroptosis, thereby collectively shaping either immunosuppressive or antitumor phenotypes within the TIME.

Lymphoid cells of the immune system, including most B cells, T cells, and NK cells, can undergo malignant transformation during development or differentiation. Different DLBCL subtypes arise from distinct B cell states. Using EcoTyper and Wishbone model analysis, the authors identified five B cell states (S1–S5) and reported that GCB-DLBCL originated from S1 (GC-like B cells), whereas ABC-DLBCL primarily arose from S2–S5, representing intermediate stages following S1 but prior to full differentiation into memory B cells or plasmablasts [60]. 

**Figure 3:**
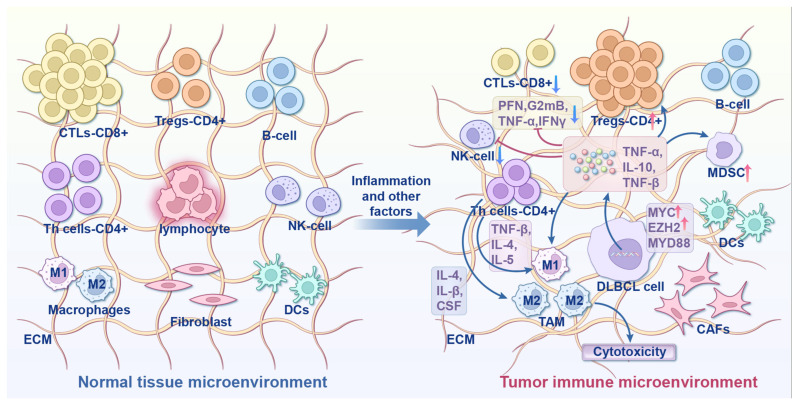
Formation of the tumor immune microenvironment induced by adverse factors such as inflammatory cytokines in the normal tissue microenvironment. Under normal conditions, the tissue microenvironment maintains immune homeostasis through coordinated interactions among its components. In the TIME, CD8^+^ T cells are driven to exhaustion due to suppression by the TGF-β and PD-L1/PD-1 pathways, while immunosuppressive Treg CD4^+^ T cells increase. Tumor-associated macrophages (TAMs) and myeloid-derived suppressor cells (MDSCs) are recruited and enriched by the tumor, inhibiting T cell function through mechanisms including ROS production. The extracellular matrix (ECM), secreted by stromal cells, becomes disorganized, thereby facilitating tumor cell survival, proliferation, and immune evasion. Tumor cells release chemokines such as TGF-β and IL-6 to recruit immune cells and secrete lactate to suppress immune function, thereby shaping an immunosuppressive microenvironment conducive to their survival. Immune and stromal cells undergo functional polarization—shifting from anti-tumor to pro-tumor phenotypes—under the influence of tumor-derived signals, forming a vicious cycle that promotes tumor progression.

Traditionally, tumor-intrinsic alterations were assumed to drive DLBCL development and drug resistance. Later, researchers recognized the direct impact of the tumor immune microenvironment (TIME) in DLBCL progression. The TIME comprises macrophages, T cells, and fibroblasts. Its functional state is dynamically regulated by lipid metabolism and ferroptosis, forming a complex metabolic-immune interaction network. The complexity of the TME arises from intracellular heterogeneity, dynamic cell-to-cell interactions, and physical and chemical barriers, together constituting an evolving ecosystem that supports tumor growth and poses a major challenge in cancer therapy [61] (Figure 3).

### 4.1. Macrophage Polarization and Functional Regulation

Macrophages are a key component of the TIME, and their polarization influences DLBCL prognosis. NR1H3, which encodes the LXR-α subtype, serves as a marker of macrophage function and predicts patient outcomes. It is highly expressed in pro-inflammatory M1 macrophages and associated with significantly prolonged patient survival; moreover, it correlates with differences in macrophage gene expression profiles (GEPs) [62].

The metabolic profile of macrophages determines their immune function. M2 macrophages create an immunosuppressive microenvironment by releasing IL-10, TGF-β, and other cytokines [62]. Macrophage membrane-encapsulated liposomes can provide targeted delivery of Vonoprazan (a potassium-competitive acid blocker), which inhibits DLBCL by disrupting mitochondrial OXPHOS and shows a synergistic effect in combination with doxorubicin [39]. This approach suggests a strategy for designing targeted delivery systems that exploit macrophage characteristics. Recent studies have also shown that glycosaminoglycan (GAG)-driven lipoprotein uptake is an important pathway for tumor cells to resist ferroptosis. Tumor cells bind lipoproteins through sulfated GAGs on their surface, facilitating the uptake of α-tocopherol (vitamin E) to inhibit lipid peroxidation. Disruption of GAG synthesis reduces lipoprotein uptake, enhances ferroptosis sensitivity, and inhibits tumor growth. In DLBCL, this mechanism may reflect metabolic competition between macrophages and tumor cells. For instance, M2 macrophages can promote tumor cell resistance to ferroptosis by secreting lipoproteins and simultaneously inhibit T cell function, thus forming a metabolic-immune synergistic suppression network [63].

### 4.2. T-Cell Function and Immune Escape

The metabolic status of T cells directly affects their anti-tumor activity. Cholesterol efflux inhibits the cytotoxicity of CD8^+^ T cells in DLBCL [33]. However, lipid peroxidation products (such as 4-HNE) released during ferroptosis can impair CD8^+^ T cell function and promote immune escape [64]. In addition, *IRF8* mutations inhibit CD4^+^ T cell activation by downregulating CD74 and HLA-DM, which are involved in MHC class II antigen processing. These mutations lead to a reduction in CD4^+^ and CD8^+^ T cells and an increase in regulatory T cells in the TME, revealing a novel mechanism of epigenetic mutation-mediated immune escape [65].

Activation of immune checkpoint molecules is another pathway for immune escape in DLBCL. In primary refractory DLBCL, C1QB^+^ macrophages inhibit CD8^+^ T cell cytotoxicity via cholesterol efflux while enhancing interactions with immune checkpoint molecules, such as LGALS9-HAVCR2 and CTLA4-CD86, leading to resistance to CAR-T cell therapy [33]. Single-cell sequencing further revealed significantly enhanced ligand-receptor interactions associated with lipid metabolism (such as AFR1-FAS) between macrophages and CD8^+^ T cells [33]. These findings suggest that combining targeted metabolic pathway interventions with immune checkpoint blockade may help reverse immunotherapy resistance.

### 4.3. Regulation of TME by Metabolites

Lipid metabolites are key regulators of the TIME. Aberrant FA metabolism can influence immune cell infiltration by altering cytokine secretion. FASN-mediated FAS promotes M2 macrophage polarization, while PUFAs inhibit T cell proliferation [28]. Endogenous ferroptosis-associated signaling molecules, such as HMGB1, induce macrophage transformation toward immunosuppressive phenotypes via the TLR4 pathway, creating a feedback loop linking ferroptosis and immunosuppression [64]. 

## 5. Prognostic Biomarkers and Their Clinical Application

Molecular markers based on lipid metabolism, iron death, and TIME provide an important framework for guiding DLBCL treatment and include monogenic, polygenic, and metabolite markers. Table 1 summarizes prognostic biomarkers related to lipid metabolism, ferroptosis, and the tumor immune microenvironment, to guide the future exploration of their underlying mechanisms and the development of targeted therapeutic strategies.

### 5.1. Lipid Metabolism-Related Markers

Aberrant expression of lipid metabolism-related genes predicts prognosis in DLBCL. *MYC* target genes are important prognostic biomarkers, with overexpression or isolated MYC rearrangements, as well as BCL2/BCL6 rearrangements, strongly associated with poor outcomes [67]. A study established a prognostic model based on 19 lipid metabolism–related genes, including fatty acid binding protein 4 (FABP4), B-cell lymphoma 6 (BCL6), and matrix metalloproteinase 9 (MMP9). These genes encompass key regulators involved in lipid transport, lipid signaling modulation, and tumor invasion, enabling precise stratification of patients with diffuse large B-cell lymphoma (DLBCL) into high-risk and low-risk groups. Patients in the high-risk group exhibited aberrant activation of the MYC and E2F signaling pathways, which on the one hand accelerates cell-cycle progression and promotes uncontrolled tumor cell proliferation, and on the other hand disrupts lipid anabolic metabolism, leading to impaired lipid homeostasis. Collectively, these alterations are associated with unfavorable clinical outcomes. These findings highlight a potential mechanism by which dysregulated lipid metabolism and aberrant cell-cycle control cooperate to drive DLBCL progression [69]. Additionally, the serum levels of triglycerides (TG), low-density lipoprotein cholesterol (LDL-C), high-density lipoprotein cholesterol (HDL-C), apolipoprotein A-I (ApoA-I), and apolipoprotein B (ApoB) were significantly lower in DLBCL patients compared to healthy controls and increased after chemotherapy. ApoA-I was identified as an independent prognostic factor. An IPI-A scoring system combining ApoA-I with the International Prognostic Index (IPI) more accurately predicted OS and progression-free survival (PFS) [73]. High HADHB expression was identified as an independent poor prognostic factor (*p* = 0.001) [29], whereas high FASN expression was associated with reduced event-free survival (EFS) in patients with double-expressing lymphoma (DEL) [20]. These molecules may serve as prognostic markers of dysregulation of lipid metabolism in DLBCL. 

Metabolite markers also have potential prognostic value. Recent studies have shown that elevated serum glutamine levels in DLBCL patients are associated with poor outcomes, whereas α-KG levels are inversely correlated with tumor aggressiveness [41]. In R-CHOP-resistant tumors, phosphatidylinositol and sphingomyelin fragments were enriched, ATP levels decreased, and AMP (adenosine monophosphate) levels increased, providing new insights into mechanisms of drug resistance and efficacy monitoring [35].

### 5.2. Ferroptosis-Related Markers

The expression of genes regulating ferroptosis is strongly correlated with the prognosis of DLBCL. *SH3GL1* is a key gene encoding endopeptide A2, affecting proliferation and survival of DLBCL cells by inhibiting FTH1-mediated ferritin autophagy and iron death [55]. High expression of KMT2D improves prognosis by enhancing ferroptosis [83]. High expression of PRDX1/2 may indicate reduced susceptibility of DLBCL to ART [54]. In addition, morphological features of ferroptosis, such as cell membrane blebbing, can be detected by deep convolutional neural networks, which significantly correlate with lipid peroxidation levels, providing a novel approach for clinical assessment of sensitivity to ferroptosis [90].

Prognosis also varies among DLBCL subtypes. High TCP1 expression in GCB subtypes is associated with increased sensitivity to ferroptosis and better prognosis, whereas high TCP1 expression in non-GCB subtypes correlates with poor prognosis [57]. Similarly, high BRD4 expression in GCB subtypes predicts potential responsiveness to BET inhibitors combined with ferroptosis inducers [59]. Furthermore, the activity of ferroptosis-related pathways can be assessed using combined molecular markers. For instance, the ACSL4/GPX4 expression ratio may reflect ferroptosis sensitivity, with a higher ratio indicating better prognosis [28]. These molecular markers may help refine prognostic stratification based on ferroptosis.

### 5.3. TIME-Related Markers

High expression of NR1H3, a marker of M1-type macrophages, is associated with longer survival in patients with DLBCL and is independent of traditional prognostic factors [62]. Recent studies suggest that CXCL9, CCL18, C1QA, and CTSC are prognostic biomarkers associated with the TIME [84]. In addition, an increased proportion of C1QB^+^ macrophages in the TME is strongly associated with resistance to CAR-T therapy [33]. *IRF8* mutations are associated with abnormal immune cell infiltration and may serve as a predictor of immune escape [65].

Drug resistance remains a major challenge in contemporary oncology, significantly limiting the clinical efficacy of chemotherapy, targeted therapy, and immunotherapy. Dynamic remodeling of the TME plays a central role in driving therapy resistance. Currently, multi-omics approaches integrating genomic, epigenomic, transcriptomic, proteomic, and metabolomic data have revealed key mechanisms underlying resistance [91,92]. Models incorporating lipid metabolism-related genes with features of immune cell infiltration can more accurately predict patient responses to immunotherapy [69] and provide a foundation for personalized treatment. 

## 6. Treatment Strategies

Based on these mechanistic insights, current targeted strategies addressing lipid metabolism, ferroptosis, and the TIME primarily involve single-target agents, combination therapies, and innovative delivery systems in Table 2.

### 6.1. Lipid Metabolism-Targeted Therapy

Inhibitors of FA metabolism and novel combination therapy strategies are current research hotspots. Based on the central role of FA metabolism, the combination of ranolazine, an FAO inhibitor, with an mTOR inhibitor significantly enhances cytotoxicity in DLBCL cells [30]. Ranolazine can selectively kill DLBCL cells with high HADHB expression [29]. FASN inhibitors, such as TVB-2640, may also potentiate DLBCL cell death when used in combination with mTOR inhibitors [21,28]. Although orlistat has not been approved for clinical cancer therapy, preclinical studies have demonstrated its inhibitory activity against fatty acid synthase (FASN), providing a reference for its potential consideration as a candidate agent for lipid metabolism–targeted therapy in diffuse large B-cell lymphoma (DLBCL) [106].

Recent studies have shown that targeting the YTHDF2-ACER2-ceramide metabolic axis can inhibit DLBCL cell growth. Drugs, such as Ibrutinib and Venetoclax, enhance the efficacy of existing therapies by targeting YTHDF2 or ACER2 to restore ceramide levels [40]. Modulators of cholesterol metabolism also contribute to DLBCL treatment. A retrospective study showed that, compared with DLBCL patients treated with R-CHOP alone, those receiving concomitant statin therapy achieved a higher complete response (CR) rate and a longer progression-free survival (PFS). Statins combined with TSN enhance anti-tumor activity [32], whereas SYK inhibitors suppress BCR-dependent DLBCL by blocking cholesterol synthesis [31]. 

### 6.2. Application of Ferroptosis Inducers

Ferroptosis inducers exhibit subtype-specific efficacy in DLBCL. Liposome-encapsulated RSL3, a ferroptosis inducer enriched with PUFAs, enhances targeted delivery and increases susceptibility to ferroptosis in GCB-DLBCL cells, reducing IC_50_ values by 7.1- to 14.5-fold compared with free RSL3 [107]. The cytotoxic effect of RSL3 is greater in GCB subtypes than in non-GCB subtypes [57]. Imidazolone (IKE), a potent and metabolically stable ferroptosis inducer, triggers ferroptosis in DLBCL by inhibiting system xc-, and its toxicity can be mitigated through nano-delivery systems [96]. 

Recent studies have highlighted the coordinated effects of multiple ferroptosis inducers in DLBCL treatment. ART could induce ferroptosis by targeting PRDX1/2 and significantly inhibits tumor growth in U2932 xenograft models without notable hepatotoxicity or nephrotoxicity [54]. BET inhibitors combined with RSL3 or dimethyl fumarate exert synergistic cytotoxicity in GCB-subtype DLBCL, achieving over 60% tumor volume reduction in animal models [59]. Additionally, CRISPR/Cas9-mediated knockout of the *SH3GL1* gene restores DLBCL cell susceptibility to ferroptosis and overcomes doxorubicin resistance, providing a potential target for future drug development aimed at mitigating chemoresistance [55]. 

Currently, combination therapy strategies show considerable promise. For instance, CISD2 silencing combined with ferroptosis inducers [53] and α-KG combined with chemotherapeutic agents [41] can enhance antitumor efficacy. In addition, interference with GAG synthesis blocks lipoprotein-mediated ferroptosis resistance, and its combination with RSL3 may further improve therapeutic outcomes in DLBCL [63]. 

### 6.3. TIME-Targeted Strategies

Therapeutic approaches targeting the TIME, including monoclonal antibodies, immune checkpoint inhibitors, and chimeric antigen receptor (CAR) T-cell therapy, have been approved or are under investigation for aggressive BCL. Recently developed monoclonal antibodies, such as glofitamab, mosunetuzumab, and epcoritamab, have demonstrated efficacy in DLBCL by simultaneously engaging CD20 on tumor cells and CD3 on T cells to activate cytotoxic responses [97,98,99]. Checkpoint inhibitors, including pembrolizumab and atezolizumab, can be combined with ibrutinib to alleviate immunosuppression in the TME, restore CD8^+^ T-cell cytotoxicity, remodel TIME, and enhance antitumor activity [100]. Additionally, CAR-T cell therapy has emerged as an important treatment modality in DLBCL. For example, tisagenlecleucel reprograms autologous T cells to express a CD19-targeting CAR, enabling specific recognition and elimination of CD19^+^ BCL [105]. 

In addition to emerging therapeutic strategies, conventional agents targeting key signaling pathways in DLBCL, such as DNA methyltransferase inhibitors (DNMTis) and drugs addressing MYC, BCL2, or BCL6 rearrangements, have demonstrated promising efficacy [101,102,103,104]. Furthermore, the combination of metformin and L-asparaginase enhances the response of DLBCL to immunotherapy by disrupting lipid metabolism and antioxidant defenses [108]. Novel delivery systems are also being explored; for instance, nucleolin-targeted silicon-based nanoparticles co-loaded with doxorubicin (DOX) and indocyanine green (ICG) induce apoptosis through synergistic chemotherapy and sonodynamic therapy (SDT), markedly suppressing tumor growth in animal models [109]. Collectively, these findings suggest that targeting metabolic-immune interactions and remodeling the TME through multi-targeted and multimodal strategies holds considerable promise for the treatment of DLBCL. 

### 6.4. Subtype-Specific Treatment Strategies

The molecular subtypes of DLBCL exhibit significant differences in lipid metabolism, sensitivity to ferroptosis, and the tumor immune microenvironment, which directly influence the selection of treatment strategies. The GCB subtype demonstrates higher sensitivity to ferroptosis inducers, and lipid metabolism-targeted drugs may hold greater potential in this subtype [110]. The ABC subtype is often associated with NF-κB pathway activation, responds better to BCR pathway inhibitors and immune checkpoint blockers, while ferroptosis inducers show limited efficacy in this subtype [111]. Although the PMBL subtype is relatively rare, its distinct metabolic and immune characteristics suggest potential specific responses to cholesterol metabolism modulators and CAR-T therapy [112]. Therefore, future treatment strategies should be individually tailored based on subtype-specific metabolic and immune features to enhance efficacy and reduce drug resistance.

### 6.5. Challenges in Clinical Translation

Nevertheless, the translation of these therapeutic strategies into clinical practice still faces multiple challenges and risks. A primary concern is off-target toxicity. Both lipid metabolism and ferroptosis-related pathways play crucial physiological roles in normal tissues such as the liver, kidneys, and nervous system. Broad inhibition of these pathways may therefore lead to significant toxicities—for example, ferroptosis inducers may damage normal cells, while FASN inhibitors could disrupt physiological lipid synthesis [113,114]. Secondly, tumor cells exhibit remarkable metabolic plasticity and redundancy. When a specific lipid synthesis or uptake pathway is inhibited, cancer cells can activate compensatory mechanisms to sustain survival, such as enhancing exogenous uptake when fatty acid synthesis is blocked or activating parallel defense systems like FSP1–CoQ10 upon GPX4 inhibition. Such redundancy frequently leads to resistance to monotherapy [115]. Furthermore, DLBCL displays considerable inter- and intra-tumoral heterogeneity. Significant differences in metabolic dependencies and ferroptosis sensitivity exist not only between molecular subtypes and genetic backgrounds but also among different clones within the same tumor [116]. Therefore, future treatment strategies must be guided by reliable biomarkers for patient stratification, enabling truly individualized and precision therapeutic intervention.

## 7. Conclusions

The regulatory network linking lipid reprogramming, ferroptosis, and TIME in DLBCL shapes both tumor progression and therapeutic response. Aberrant lipid metabolism not only fuels DLBCL cell proliferation but also influences ferroptosis susceptibility by modulating molecules, such as GPX4, while metabolites derived from lipid metabolism remodel the TIME. Activation or inhibition of ferroptosis regulates immune cell function through DAMPs and lipid peroxidation products, whereas immune cells within the TIME respond to tumor metabolism and cell death pathways through metabolic competition and cytokine secretion. Biomarkers arising from these processes provide new tools for prognostic stratification in DLBCL, and combination strategies targeting these pathways have significant clinical implications.

Importantly, metabolic features as biomarkers for stratifying patients with diffuse large B-cell lymphoma hold substantial translational potential and merit focused investigation. This concept represents one of the core innovative application directions of this review, which integrates lipid metabolism, ferroptosis, and the tumor immune microenvironment. For instance, lipid metabolism–related gene signatures can serve as powerful tools for the precise identification of patient populations likely to respond to specific therapies. Gene models constructed based on lipid metabolism–associated genes such as FABP4, BCL6, and MMP9 not only enable effective risk stratification of DLBCL patients but also facilitate the identification of subgroups that are sensitive to fatty acid synthase (FASN) inhibitors, thereby providing a rational basis for the development of personalized therapeutic strategies. Moreover, serum lipid metabolites and lipid metabolism–related genes, when used as independent prognostic factors, can be integrated with traditional clinical indices such as the International Prognostic Index (IPI) to further refine patient stratification systems. This integrated approach improves the accuracy of therapeutic response prediction and enhances the precision of metabolism-targeted therapies in DLBCL.

However, the high heterogeneity of DLBCL remains a major challenge, with marked differences between subtypes in response to metabolic interventions and ferroptosis induction, necessitating the development of subtype-specific treatment regimens. The specific mechanisms underlying metabolism-immune interactions, such as how lipid metabolites precisely regulate the expression of immune checkpoint molecules, are not fully understood yet. In addition, the clinical utility of biomarkers needs to be validated in larger prospective studies. Future research should integrate approaches, such as single-cell sequencing, spatial metabolomics, and analyses of the TIME in DLBCL, to develop more precise targeted strategies and improve patient outcomes.

## Figures and Tables

**Table 1:** Prognostic biomarkers associated with lipid metabolism, ferroptosis, and the tumor immune microenvironment.

	Prognostic Biomarker	Effect	Ref.
Lipid Metabolism	FASN	High FASN expression is an independent predictor of poor prognosis.	[20,66]
	MYC	MYC rearrangement or overexpression confers an adverse prognosis in DLBCL.	[67,68]
	E2F pathway activation	E2F activation promotes tumor aggressiveness and predicts poor prognosis in DLBCL.	[69,70,71,72]
	LDL-C	High levels of LDL-C correlate with a lower rate of complete response (CR) and a higher risk of disease progression.	[73,74,75]
	HDL-C	High levels of HDL-C are associated with a reduced risk of progression.	[76,77,78]
	ApoA1	Low levels of ApoA1, a major HDL structural protein, are an independent poor prognostic factor for overall survival (OS).	[79]
	APOC1	High levels are associated with a poor prognosis.	[80]
	19-gene lipid metabolism prognostic signature (TMEM176B, LAYN, RAB6B, etc.)	A 19-gene risk model was developed; a high risk score derived from these genes was significantly associated with poorer overall survival (OS).	[69]
	HADH	Low HADHA/HADHB expression associates with poor prognosis.	[29,81]
	Cytoplasmic Lipid Droplets	predicts poor prognosis.	[55,66]
	Adipophilin	It predicts poor prognosis and serves as a surrogate marker for lipid droplets via its role as a coat protein.	[55]
Ferroptosis	FTH1	It mediates ferritinophagy and ferroptosis, cooperates with SH3GL1, and influences chemoresistance and prognosis.	[55]
	SH3GL1	It inhibits FTH1-mediated ferritinophagy and ferroptosis; its high expression is linked to poor prognosis.	[55]
	GPX4	A key ferroptosis inhibitor; low levels confer sensitivity to inducers.	[82]
	ACSL4	Promotes synthesis of peroxidation-prone phospholipids; increases ferroptosis sensitivity.	[57]
	TCP1	Promotes ferroptosis by stabilizing ACSL4; high expression in the GCB subtype predicts better prognosis.	[57]
	KMT2D	The most frequently mutated gene in DLBCL; a key driver and prognostic marker.	[83]
	PRDX1/PRDX2	Scavenges ROS; its high expression inhibits ferroptosis and is linked to poor prognosis. High expression links to anti-tumor M1 macrophages and improved survival.	[54,62]
Tumor Immune Microenvironment	NR1H3		
	CXCL9		
	CCL18	High expression is associated with poor prognosis.	[84]
	C1QA	Elevated C1QA predicts adverse outcomes.	[84]
	CTSC (Cathepsin C)	High expression is associated with poor prognosis.	[84]
Tumor Immune Microenvironment	C1QB+	High expression is associated with a favorable prognosis.	[33]
	CD58 mutation	Immune synapse costimulation is frequently mutated, which can lead to tumor immune evasion.	[85]
Other core biomarkers	FAT4	Serves as a prognostic biomarker for the elderly subgroup	[86]
	ETV6	Independently predicts shorter PFS	[87]
	STAT3	High p-STAT3 predicts poor OS and PFS.	[88]
	MYD88/CD79B mutation	High expression is associated with poor prognosis.	[86]
	BEND4	High expression is associated with poor prognosis.	[89]

**Table 2:** Current treatment strategies for DLBCL.

	Drug	Combined Medication	Target/Pathway	Mechanism/Effect	Ref.
Lipid metabolism Ferroptosis	GLS1 inhibitor CB-839	BCL2 inhibitor ABT-199	Glutamine catabolic metabolism	Can induce the production of a large amount of reactive oxygen species, exhibiting cytotoxicity.	[93]
	Atorvastatin	R-CHOP	HMG-CoA reductase	Reduce intracellular cholesterol levels and induce apoptosis in tumor cells.	[75,94]
	FAO inhibitor 4-BrCA	Metformin/L-Asparaginase	Fatty acid oxidation	Inhibit key enzymes of β-oxidation, blocking energy supply.	[30]
	FASN inhibitor C75	Metformin/L-Asparaginase	Fatty acid synthesis	Inhibit de novo synthesis of fatty acids, affecting membrane structure and signaling pathways.	[30]
	mTOR pathway inhibitor Rapamycin	FASN inhibitor C75	mTOR pathway	Induce metabolic crisis by inhibiting the electron transport chain or depleting amino acids.	[30]
	Ibrutinib/Venetoclax	/	YTHDF2–ACER2–Ceramide Metabolism Axis	Inhibiting YTHDF2 and reducing ACER2 lead to the accumulation of ceramides, decreased S1P, and the inhibition of the PI3K/AKT and ERK pathways, thereby suppressing tumor growth.	[40]
	Toosendanin	Statins	Upregulate cholesterol biosynthesis; inhibit the PI3K/Akt signaling pathway and PLK1.	Inhibit the PI3K/Akt signaling pathway, downregulating the key PI3K/Akt survival signaling pathway, leading to the death of cancer cells; inhibit Polo-like kinase 1 (PLK1), resulting in cell cycle arrest.	[32]
	SYK inhibitor R406/ Fostamatinib (R788)	/	Inhibition of key kinases in the B cell receptor (BCR) signaling pathway	Inhibiting SYK will block the transmission of downstream survival signals such as PI3K/AKT and NF-κB.	[31]
	MCT1 small molecule inhibitor (AZD3965)	Doxorubicin, rituximab, CB-839	Inhibiting SYK will block the transmission of downstream survival signals such as PI3K/AKT and NF-κB.	Inhibition of tumor cell lactate efflux, resulting in intracellular lactate accumulation, glycolytic inhibition, and suppression of tumor growth.	[95]
Ferroptosis	Artesunate	/	PRDX1/PRDX2	ART binding to PRDX1 (Gly4) and PRDX2 (Arg7, Thr120), inhibiting their enzymatic activity and triggering ferroptosis.	[54]
	BET inhibitor	Dimethyl fumarate/RSL3	BRD4 inhibition	BRD4 inhibition downregulates FSP1 expression, disabling the cellular ferroptosis defense and sensitizing cells to ferroptosis inducers.	[59]
	Imidazolidone Irastin	/	system xc- inhibition	Inhibition of system xc- prevents cystine uptake, leading to glutathione depletion, lipid peroxidation, and ferroptosis.	[96]
Tumor Immune Microenvironment	Glofitamab	/	CD20, CD3	Bispecific binding to CD20 and CD3 constructs an immune synapse, activating cytotoxic T cells for tumor killing.	[97]
	Mosunetuzumab	R-CHOP	CD20, CD3	A bispecific T cell engager that activates immune-mediated tumor killing.	[98]
	Epcoritamab	/	CD20, CD3	Recruits T cells to tumor cells and activates them for killing.	[99]
	Pembrolizumab, Atezolizumab	Ibrutinib	PD-1/PD-L1 inhibition	Relief of immunosuppression, restoration of CD8^+^ T cell cytotoxicity, and immune microenvironment remodeling.	[100]
Tumor Immune Microenvironment	Chidamide	Venetoclax/R-CHOP	MYC, BCL2, TP53	Inhibits DLBCL growth by downregulating the transcription and translation of MYC and TP53, and upregulating the expression of the pro-apoptotic protein BIM.	[101,102]
	Decitabine	RDHAP	DNA Methyltransferase	Inhibition of tumor growth via DNMT suppression and subsequent demethylation of tumor suppressor gene promoters, restoring apoptosis and differentiation.	[103]
	Lenalidomide	ViPOR	Key survival signaling pathways in DLBCL	Activates the immune system against tumors via immunomodulation and immune-mediated cytotoxicity.	[104]
	Tisagenlecleucel	Lymphodepleting Chemotherapy	/	Targets and kills CD19^+^ tumors via infusion of genetically modified, CD19 CAR-expressing T cells.	[105]

## Data Availability

No new data were created or analyzed in this study.

## Abbreviations

The following abbreviations are used in this manuscript:

AA	Arachidonic Acid
ABC-DLBCL	Activated B-Cell-like Diffuse Large B-Cell Lymphoma
ACLY	ATP-citrate Lyase
ACSL4	Acyl-CoA Synthetase Long-Chain Family Member 4
ADM	Adriamycin
ApoA-I	Apolipoprotein A-I
ApoB	Apolipoprotein B
ART	Artesunate
BCL2	B-cell Lymphoma 2
BCL6	B-cell Lymphoma 6
BCR	B-cell Receptor
BETi	BET Inhibitor
BRD4	Bromodomain-containing Protein 4
CAR-T	Chimeric Antigen Receptor T-cell
CISD2	CDGSH Iron-Sulfur Domain 2
COO	Cell-of-Origin
CR	Complete Response
DAG	Diacylglycerol
DEL	Double-Expressor Lymphoma
DLBCL	Diffuse Large B-Cell Lymphoma
DAMPs	Damage-Associated Molecular Patterns
DNMTi	DNA Methyltransferase Inhibitor
DOX	Doxorubicin
E2F	E2F Transcription Factor
ECM	Extracellular Matrix
EFS	Event-Free Survival
eIF4B	Eukaryotic Initiation Factor 4B
FA	Fatty Acid
FABP4	Fatty Acid Binding Protein 4
FAO	Fatty Acid Oxidation
FAS	Fatty Acid Synthesis
FASN	Fatty Acid Synthase
FSP1	Ferroptosis Suppressor Protein 1
FTH1	Ferritin Heavy Chain 1
FPN	Ferroportin
GAG	Glycosaminoglycan
GCB-DLBCL	Germinal Center B-cell-like Diffuse Large B-Cell Lymphoma
GCIB-SIMS	Gas Cluster Ion Beam Secondary Ion Mass Spectrometry
GEP	Gene Expression Profiling
GPX4	Glutathione Peroxidase 4
GSH	Glutathione
HADHA/HADHB	Hydroxyacyl-CoA Dehydrogenase Trifunctional Multienzyme Complex Subunit Alpha/Beta
HDACi	Histone Deacetylase Inhibitor
HDL-C	High-Density Lipoprotein Cholesterol
HMOX1	Heme Oxygenase 1
ICG	Indocyanine Green
IC50	Half Maximal Inhibitory Concentration
IL-6/IL-10	Interleukin-6/10
IKE	Imidazole Ketone Erastin
IONs	Iron Oxide Nanoparticles
IPI	International Prognostic Index
KDACi	Lysine Deacetylase Inhibitor
KMT2D	Lysine Methyltransferase 2D
LDL-C	Low-Density Lipoprotein Cholesterol
LIP	Labile Iron Pool
LPCAT3	Lysophosphatidylcholine Acyltransferase 3
LXR-α	Liver X Receptor Alpha
MDSCs	Myeloid-Derived Suppressor Cells
MHC	Major Histocompatibility Complex
MMP9	Matrix Metalloproteinase 9
mTOR	Mechanistic Target of Rapamycin
MYC	MYC Proto-Oncogene
NF-κB	Nuclear Factor Kappa B
NHL	Non-Hodgkin Lymphoma
NRF2	Nuclear Factor Erythroid 2–Related Factor 2
OS	Overall Survival
OXPHOS	Oxidative Phosphorylation
PD-1/PD-L1	Programmed Cell Death Protein 1/Programmed Death-Ligand 1
PDK1	3-Phosphoinositide-Dependent Protein Kinase 1
PFS	Progression-Free Survival
PI3K	Phosphatidylinositol 3-Kinase
PIP2/PIP3	Phosphatidylinositol 4,5-Bisphosphate/3,4,5-Trisphosphate
PKC/PKD	Protein Kinase C/Protein Kinase D
PLK1	Polo-like Kinase 1
PMBL	Primary Mediastinal B-cell Lymphoma
PRDX1/2	Peroxiredoxin 1/2
PTEN	Phosphatase and Tensin Homolog
PTGDS	Prostaglandin D2 Synthase
PUFAs	Polyunsaturated Fatty Acids
R-CHOP	Rituximab, Cyclophosphamide, Doxorubicin, Vincristine, Prednisone
ROS	Reactive Oxygen Species
RSL3	RSL3 (ferroptosis inducer)
SDT	Sonodynamic Therapy
SH3GL1	SH3 Domain Containing GRB2 Like, Endophilin A2
SLC7A11/xCT	Solute Carrier Family 7 Member 11/Cystine/Glutamate Transporter
SMG1	SMG1, Nonsense Mediated mRNA Decay Associated PI3K Related Kinase
STAT3	Signal Transducer and Activator of Transcription 3
SYK	Spleen Tyrosine Kinase
TAMs	Tumor-Associated Macrophages
TCP1	T-complex Protein 1
TFR	Transferrin Receptor
TG	Triglycerides
TGF-β	Transforming Growth Factor Beta
TIME	Tumor Immune Microenvironment
TLR4	Toll-like Receptor 4
TME	Tumor Microenvironment
TP53/p53	Tumor Protein P53
TSN	Toosendanin
USP11	Ubiquitin-Specific Protease 11
α-KG	Alpha-Ketoglutarate
4-HNE	4-Hydroxynonenal
